# Corticomotor Excitability Changes Induced by Progressive Balance Exercises in Chronic Ankle Instability: a Randomized Clinical Trial

**DOI:** 10.1002/brb3.71222

**Published:** 2026-01-29

**Authors:** Mahdis Purzolfi, Cyrus Taghizadeh Delkhoush, Majid Mirmohammadkhani

**Affiliations:** ^1^ Department of Physical Therapy, School of Rehabilitation Sciences Semnan University of Medical Sciences Semnan Iran; ^2^ Neuromuscular Rehabilitation Research Center, Research Institute of Neurosciences Semnan University of Medical Sciences Semnan Iran; ^3^ Social Determinants of Health Research Center Semnan University of Medical Sciences Semnan Iran

**Keywords:** ankle sprain, cortical excitability, motor evoked potential, transcranial magnetic stimulation

## Abstract

**Background:**

Progressive balance exercises may change corticomotor excitability during the learning process of postural stability control. The primary purpose of the present study was to assess corticomotor excitability corresponding to the peroneus longus muscle under transcranial magnetic stimulation following 6 weeks of progressive balance exercises in individuals with chronic ankle instability.

**Methods:**

Eligible volunteers diagnosed with chronic ankle instability were randomly assigned to either the intervention group or the control group. The intervention group practiced progressive balance exercises every other day for 6 weeks, while the control group continued their daily activities. The corticomotor excitability outcome measures included the active and resting corticomotor thresholds, the motor evoked potential, and the cortical silent period of the peroneus longus muscle, which were measured using an electromyography device under a transcranial magnetic stimulator. The outcome measures were measured in the intervention group before and after progressive balance exercises, and in the control group at baseline and again after a 6‐week interval.

**Results:**

The corticomotor thresholds and cortical silent period of the peroneus longus muscle were significantly decreased within groups (*p*‐values < 0.001; *η* ^2^ *p* > 0.580). In addition, the normalized motor evoked potential of the peroneus longus muscle exhibited a significant increase within groups (*p*‐value < 0.001; *η* ^2^ *p* = 0.265). Interestingly, a significant interaction effect was revealed between the within‐group and between‐group effects for the corticomotor excitability outcome measures related to the peroneus longus muscle (*p*‐values < 0.001; *η* ^2^ *p* > 0.311).

**Conclusions:**

Six weeks of progressive balance exercises significantly increased corticomotor excitability corresponding to the peroneus longus muscle in individuals with chronic ankle instability.

## Graphical abstract

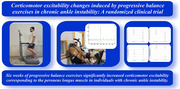

## 1 Introduction

Inversion ankle sprain is among the most common injuries in both athletic and non‐athletic populations, with an incidence rate of 2.15 per 1000 person‐years (Waterman et al. 2010). Approximately 70% of individuals with inversion ankle sprain may experience recurrent ankle sprains or episodes of ankle giving way, which are recognized as chronic ankle instability (CAI) (Hertel and Corbett 2019; Herzog et al. 2019). This chronic instability results from a complex interplay of mechanical, sensory‐perceptual, and motor‐behavioral impairments, which are interrelated components of a multifaceted condition (Hertel and Corbett 2019).

Sensory inputs transmitted to the somatosensory cortex can be partially diminished due to ankle sprains and ruptures of periarticular capsuloligamentous structures (Ward et al. 2015). These changes subsequently reorganize motor programs within the motor cortex, which adversely impact balance control on both the affected and unaffected sides (Rossini and Pauri 2000; Ward et al. 2015). This chronic neuromuscular disorder can reduce performance, particularly during challenging or complex motor tasks (Kim et al. 2019; Papegaaij et al. 2016), and negatively impact health‐related quality of life (Terada et al. 2022).

Previous studies have shown that balance exercises in individuals with CAI modify postural stability control (Youssef et al. 2018) and functional balance control (Mollà‐Casanova et al. 2021), decrease reaction time of the peroneal muscles to ankle disturbances (Plangtaisong et al. 2021), and enhance ankle proprioception (Ha et al. 2018). In addition, balance exercises can enhance the degrees of freedom for ankle movement by restoring the sensory‐motor pathways in the central nervous system (Mettler et al. 2015). Interestingly, balance exercises can reduce the incidence of ankle sprains among healthy athletes (Stanković et al. 2024).

Recent studies (McLeod et al. 2015; Nanbancha et al. 2019; Needle et al. 2013; Pietrosimone and Gribble 2012; Terada et al. 2016) have indicated that the motor cortex and corticospinal tract related to the anterior, lateral, and posterior leg muscles are suppressed in individuals with CAI, leading to a decrease in motor evoked outputs under the transcranial magnetic stimulation (TMS) in these muscles. In addition, structural changes within the central nervous centers related to balance control indicate a compromised capacity to stabilize posture and balance (Bączkowicz et al. 2017; Terada et al. 2019).

A previous study that prescribed a single session of balance exercises to healthy individuals found no significant changes in corticomotor excitability or postural stability control (Bakker et al. 2021). Conversely, studies that administered several sessions of balance exercises to healthy individuals observed a significant decrease in corticomotor excitability (Beck et al. 2007; Mouthon and Taube 2019). However, postural stability control was not assessed in these studies (Beck et al. 2007; Mouthon and Taube 2019).

To our knowledge, no previous studies have explored the effects of progressive balance exercises practiced over several weeks on corticomotor excitability in individuals with CAI. Previously, Chung et al. (2023) administered a single session of balance exercises to individuals with CAI and reported no significant changes in corticomotor excitability related to the soleus muscle as well as postural stability control.

To enhance postural stability control and prevent recurrent ankle sprains mediated by the sensorimotor system, progressive balance exercises extending over several weeks should be integrated into the physical therapy guidelines for individuals suffering from CAI (Sefton et al. 2011). In addition, the peroneal muscles have been identified as primary protective muscles against external and internal disturbances exerted on the ankle (Needle et al. 2013). Therefore, changes in corticomotor excitability specifically related to the peroneal muscles, compared to the anterior or posterior leg muscles, may predominantly affect postural stability control.

To our knowledge, the precise impact of progressive balance exercises extending over several weeks on corticomotor excitability related to the peroneus longus muscle, a major protective muscle, remains unclear. It was hypothesized that 6 weeks of progressive balance exercises significantly modulate corticomotor excitability corresponding to the peroneus longus muscle. Therefore, the main purpose of the present study was to assess corticomotor excitability related to the peroneus longus muscle under TMS following 6 weeks of progressive balance exercises in individuals diagnosed with CAI. The results of the present study may reveal how the motor cortex contributes to postural stability control following progressive balance exercises.

## 2 Methods

### 2.1 Study Design

The current study was a controlled clinical trial involving two parallel groups of individuals diagnosed with CAI. The Research Ethics Committee of Semnan University of Medical Science approved the present study (IR.SEMUMS.REC.1401.249). The present study was subsequently registered in the Iranian Clinical Trials Registry (IRCT20221218056847N1).

### 2.2 Randomization and Blinding

Using the permuted block randomization method, participants were equally assigned to either the intervention group or the control group. A statistician who was blinded to both the evaluation procedure and the intervention protocol randomly arranged distinct sequences of four‐unit blocks using random numbers extracted from a random number table. These blocks were then sealed in non‐transparent envelopes. To control the impact of height on nerve conduction velocity and the effect of age and sex on corticomotor excitability, the present study balanced these confounding variables across groups.

In this single‐blinded trial, a physical therapist supervised and guided the progressive balance exercises in the intervention group, while another physical therapist measured the outcome measures in both groups.

### 2.3 Participants

Volunteers, aged between 18 and 60 years (Fisher et al. 2016), who were diagnosed with CAI and admitted to medical centers affiliated with Semnan University of Medical Sciences, were recruited through a convenience sampling method. The present study was conducted at the Neuromuscular Rehabilitation Research Center of Semnan University of Medical Science between February and October 2023. The research method was explained to participants in plain language. Subsequently, they were requested to read and sign the informed consent form. An orthopedic surgeon and a physical therapist screened volunteers based on the inclusion and exclusion criteria.

Volunteers with a first unilateral Grade II ankle sprain in the dominant ankle within the past 1–3 years (Gribble et al. 2014) and a Cumberland Ankle Instability Tool (CAIT) score of less than 24 (Gribble et al. 2014) were included in the present study. Volunteers were required to have recurrently experienced Grade I or II ankle sprains within the past 1–3 years, with the most recent ankle sprain occurring up to three months prior to the study (Gribble et al. 2014; Hertel and Corbett 2019). In addition, they were required to report recurrent episodes of ankle giving way within the past 1–3 years, with at least two episodes occurring within the past 6 months (Gribble et al. 2014; Hertel and Corbett 2019). No upper limit was specified for episodes of ankle giving way in the present study (Hertel and Corbett 2019). The grades of mechanical instability resulting from prior ankle sprains were verified using medical records, based on special tests, including the anterior drawer test and the talar tilt test, and clinical indicators, including swelling, subcutaneous hemorrhage, and pain (Hertel and Corbett 2019; Wells et al. 2019).

Volunteers with a previous Grade III ankle sprain, a Grade I or II ankle sprain within the past 3 months, or fewer than two episodes of ankle giving‐way within the past 6 months were excluded from the present study (Gribble et al. 2014; Hertel and Corbett 2019; McLeod et al. 2015; Wells et al. 2019).

The exclusion criteria included other musculoskeletal disorders in the lower quadrant of the skeleton (Terada et al. 2016), orthopedic diseases (Terada et al. 2016), neuromuscular disorders (Gribble et al. 2014), neurological diseases (Pietrosimone and Gribble 2012), systemic diseases (Jain et al. 2016), rheumatologic diseases, balance disorders due to vestibular or visual disturbances (Kosik et al. 2017; Terada et al. 2022), medication intake within the past 2 weeks (Chung et al. 2023), and physical therapy for the lower quadrant of the skeleton within the past 6 months (Jain et al. 2016; Kosik et al. 2017). They also covered any limitations for TMS according to the guidelines provided by the National Institutes of Neurological Disorders and Stroke (Rossi et al. 2009). These guidelines included cerebral or cardiac stimulator implants, orthopedic metal implants, ocular or cochlear implants, medication pump implants, intracranial clips, intracardiac lines, drug or alcohol addiction, pregnancy or breastfeeding, cardiovascular diseases, psychiatric disorders, and neurological disorders including seizures, migraines, intracranial malignancy, and intracranial hypertension.

**FIGURE 1:**
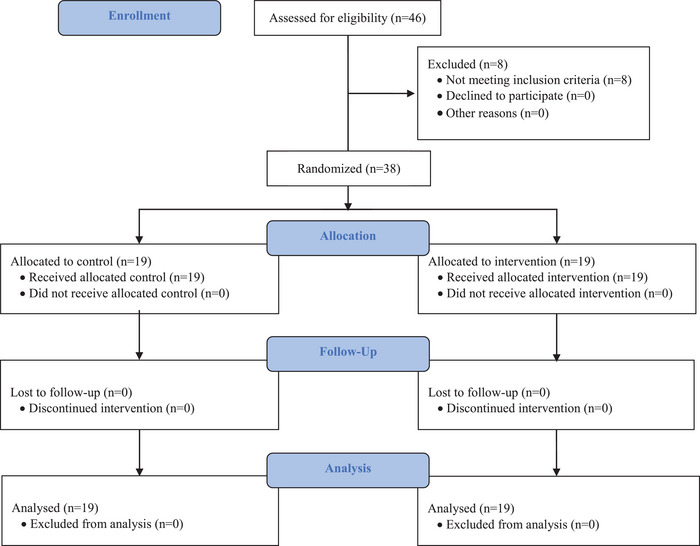
Flowchart of the present study.

The present study initially enrolled 46 volunteers, of whom eight were then excluded based on the inclusion or exclusion criteria. The study concluded with 38 volunteers (Figure 1).

The intervention group practiced progressive balance exercises every other day over 6 weeks, whereas the control group continued their regular daily activities and avoided new physical activities. The outcome measures were measured in the intervention group before and after progressive balance exercises, and in the control group at baseline and again after a 6‐week interval. To adhere to guidelines related to medical ethics, the control group also received the progressive balance exercises every other day for 6 weeks subsequent to the final assessment. To assess intra‐rater reliability, the primary outcome measures were measured twice, separated by a 2‐h interval, in five participants from each group (*N* = 10). These measurements were then repeated after 2 days.

### 2.4 Intervention

**FIGURE 2:**
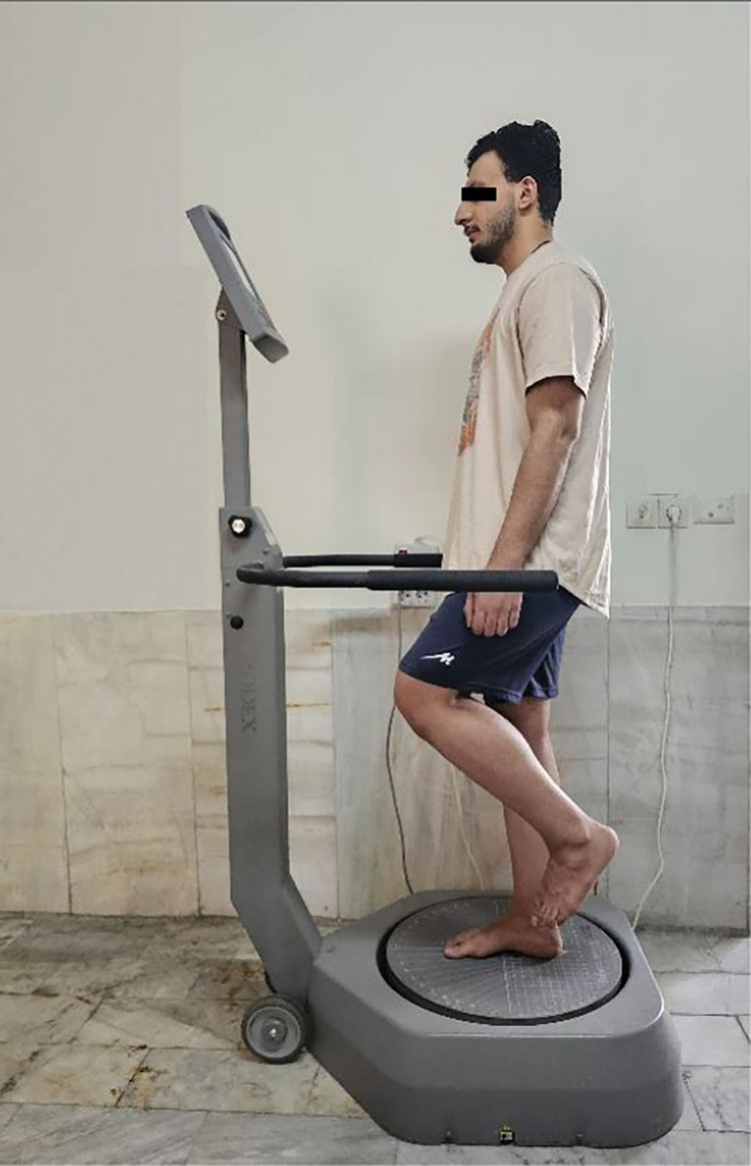
The progressive balance exercises for the intervention group and the assessment of postural stability control for both the intervention and control groups.

The intervention group received progressive balance exercises over 6 weeks, every other day (18 sessions), using the Biodex balance system (Model 950‐302, Biodex Medical Systems Inc., USA). Participants completed a 10‐min routine warm‐up and cool‐down consisting of stretching exercises targeting the lower extremity muscles before and after progressive balance exercises, respectively. The present study followed a progressive balance exercise protocol prescribed for individuals with CAI as prescribed by Jain et al. (2016), which is detailed in Table 1 and illustrated in Figure 2.

**TABLE 1:** The progressive balance exercises administered every other day from Week 1 (W_1_) to Week 6 (W_6_) for the intervention group.

Stage	State	Activity	The resistance levels opposing platform motions						Set	Duration (s)	Repetition
			W_1_	W_2_	W_3_	W_4_	W_5_	W_6_			
1	Static	Single leg stance	11	10	9	8	7	6	3	30	—
2	Static	Single leg stance	7	6	5	4	3	2	3	30	—
3	Dynamic	Anteroposterior platform tilt	7	6	5	4	3	2	3	—	6
4	Dynamic	Mediolateral platform tilt	7	6	5	4	3	2	3	—	6
5	Dynamic	Clockwise platform rotation	7	6	5	4	3	2	1	—	10
6	Dynamic	Counterclockwise platform rotation	7	6	5	4	3	2	1	—	10

Participants were requested to stand barefoot on their dominant foot centered on the platform, raise their opposite foot from the platform with slight hip flexion and 90° knee flexion, flex their dominant knee joint up to 15°, and hold their arms by their sides (Youssef et al. 2018). They were instructed to look at the display module and attempt to maintain the center of pressure on the center of the innermost circle during progressive balance exercises (Abalos and Hung 2022). In addition, they were requested to avoid lateral trunk flexion or hip abduction exceeding 30°, not to touch the platform or their dominant limb with the opposite limb, and not to grasp the support handle during the progressive balance exercises (McKeon et al. 2008; Youssef et al. 2018).

The progressive balance exercise protocol in the present study included two static exercises and four dynamic exercises, which were divided into six stages per session. Participants started at Stage 1, progressed sequentially from Stage 1–6, and completed each session at Stage 6.

Initially, participants carried out three 30‐s sets of single‐leg stance on the platform in Stages 1 and 2, each stage introducing a different level of resistance. Subsequently, they executed three sets of anteroposterior platform tilts during Stage 3 and three sets of mediolateral platform tilts during Stage 4, each set consisting of six repetitions. Lastly, they performed one set of clockwise platform rotation during Stage 5 and one set of counterclockwise platform rotation during Stage 6, each set comprising 10 repetitions. Participants were provided with a 10‐s rest following each set and a 2‐min rest following each stage.

The resistance levels opposing platform motions were adjusted at each stage based on predefined values specified weekly in Table 1. These resistance levels were progressively reduced by one level per week to provide a progressive challenge over six weeks.

### 2.5 Outcome Measures

In the present study, corticomotor excitability under TMS, including the active corticomotor threshold (ACMT), resting corticomotor threshold (RCMT), motor evoked potential (MEP), and cortical silent period (CSP), was regarded as the primary outcome measures, whereas the objective and subjective stability indices were considered secondary outcome measures.

#### 2.5.1 The Corticomotor Excitability

To avoid the excitatory effect of caffeine on corticomotor excitability, participants were requested not to consume caffeine for 12 h prior to their scheduled assessment sessions (McLeod et al. 2015). Participants were seated on an adjustable chair (Model 850‐230, Biodex Medical Systems Inc., USA) with their arms resting at their sides and were instructed to look at the screen of the Biodex multi‐joint system. In addition, their knee joints were flexed to 90° using a limb support pad, and their dominant ankles were stabilized in a neutral position on a footplate. To prevent discomfort caused by the sounds of magnetic stimulator, earplugs were inserted into their ears (McLeod et al. 2015).

The electromyographic signals were recorded using an electromyography device (Model ME6000, Mega Electronics Ltd., Finland), and processed with MegaWin software (version 3.1). The data were sampled at a frequency of 4000 Hz. To reduce skin resistance, the superolateral aspect of the leg was shaved, sanded, and cleansed with alcohol. The present study employed pre‐gelled circular Ag/AgCl surface electrodes with a 10 mm diameter. The recording electrodes with a 20 mm inter‐electrode distance were placed 2–3 cm below the fibular head on the peroneus longus muscle. These electrodes were placed at the mid‐belly of the muscle, midway between the motor end‐plate and the myotendinous junction, and in line with the muscle fibers. The ground electrode was placed on the tibial tuberosity of the dominant limb. The electromyographic signals were processed using a band‑pass filter of 1–2000 Hz (Groppa et al. 2012; Hupfeld et al. 2020).

**FIGURE 3:**
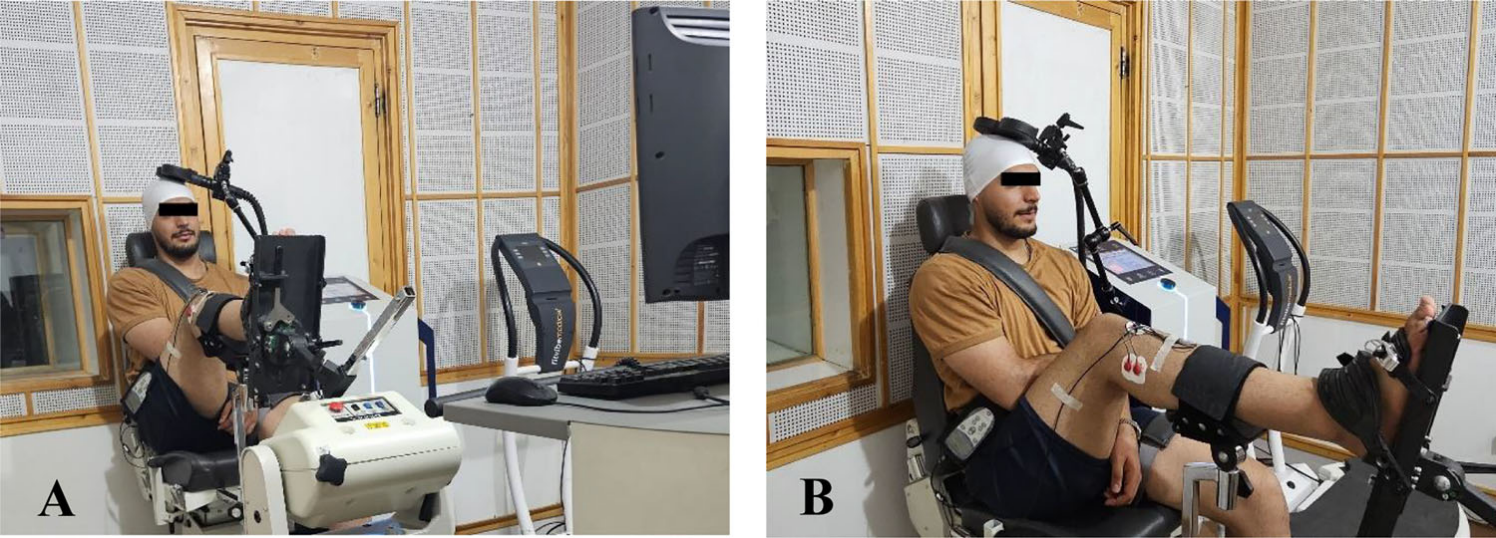
The assessment of corticomotor excitability corresponding to the peroneus longus muscle under transcranial magnetic stimulation in each group at (A) the anterior aspect and (B) the lateral aspect.

The present study utilized a magnetic stimulator (Model MagSurvePro‐100, Medina Teb Gostar Co., Iran), with a maximum output of 2 T, equipped with a planar figure‐of‐eight coil (Figure 3). To accurately locate the hotspot for the peroneus longus muscle on the motor cortex, two lines were marked on each participant's Lycra swim cap, intersecting vertically at the vertex. The midsagittal line connected the occipital protuberance to the glabella, whereas the midfrontal line crossed both external auditory meatuses (Chung et al. 2023). The figure‐of‐eight coil was oriented anteroposteriorly and was moved precisely from the vertex backward on a 1 cm × 1 cm grid outlined on the contralateral motor cortex of the dominant limb (Groppa et al. 2012; Hupfeld et al. 2020; Terada et al. 2016). The motor cortex was then stimulated at 1‐cm intervals using a single sinusoidal magnetic pulse at 50% of the maximum stimulator output, delivered at 10‐s intervals (Chung et al. 2023; Hupfeld et al. 2020; Nanbancha et al. 2019). A hotspot corresponding to the peroneus longus muscle was identified on the medial aspect of the precentral gyrus, within the interhemispheric fissure, for each participant, where the MEP consistently exhibited the maximum peak‐to‐peak amplitude (Groppa et al. 2012; Hupfeld et al. 2020; Nanbancha et al. 2019). The same hotspot was used for both the baseline and final assessments for each participant. The transcranial magnetic stimulator and the electromyography system were not synchronized in the present study. Therefore, no TMS‐related artifacts were observed in the electromyographic signal.

The minimum stimulation intensity required to evoke a peak‐to‐peak motor amplitude of 100 µV in 5 out of 10 consecutive trials during a 20% maximal voluntary isometric contraction, sustained using electromyographic biofeedback, was considered the ACMT (Groppa et al. 2012; Hupfeld et al. 2020; McLeod et al. 2015). Conversely, the minimum stimulation intensity required to evoke a peak‐to‐peak motor amplitude of 50 µV in three out of six consecutive trials during rest was regarded as the RCMT (Hupfeld et al. 2020; Lauber et al. 2018). Accordingly, the stimulation intensity was initially set to 20%–30% of the maximum stimulator output. Subsequently, it was progressively increased by 5% and then decreased by 1% to identify the minimum stimulation intensity required to evoke peak‐to‐peak motor amplitudes of 100 and 50 µV under muscle contraction and relaxation conditions, respectively (Groppa et al. 2012; Hupfeld et al. 2020). In the present study, the corticomotor thresholds are expressed as a percentage of the maximum stimulator output.

To assess the MEP and the CSP of the peroneus longus muscle, the motor cortex was stimulated across 30 trials at 120% of the AMT. To counteract neuromuscular fatigue, a 10‐s interval was maintained between stimuli (Fisher et al. 2016; Hupfeld et al. 2020).

In the present study, the peak‐to‐peak amplitude of the MEP was measured on the raw electromyographic signal. The mean peak‐to‐peak amplitude of the MEPs, measured across 30 trials, was normalized to the maximum average electromyographic amplitude recorded during the middle 1 s of three 5‐s maximal voluntary isometric contraction trials of the peroneus longus muscle (Dharia et al. 2021). To elicit maximum exertion during maximal voluntary isometric contraction trials, participants were encouraged with verbal feedback and were provided with visual feedback of their eversion torque displayed on the screen of the Biodex multi‐joint system (Dynamometer Model 900‐860 and Controller Model 835‐210, Biodex Medical Systems Inc., USA) (Dharia et al. 2021).

**FIGURE 4:**
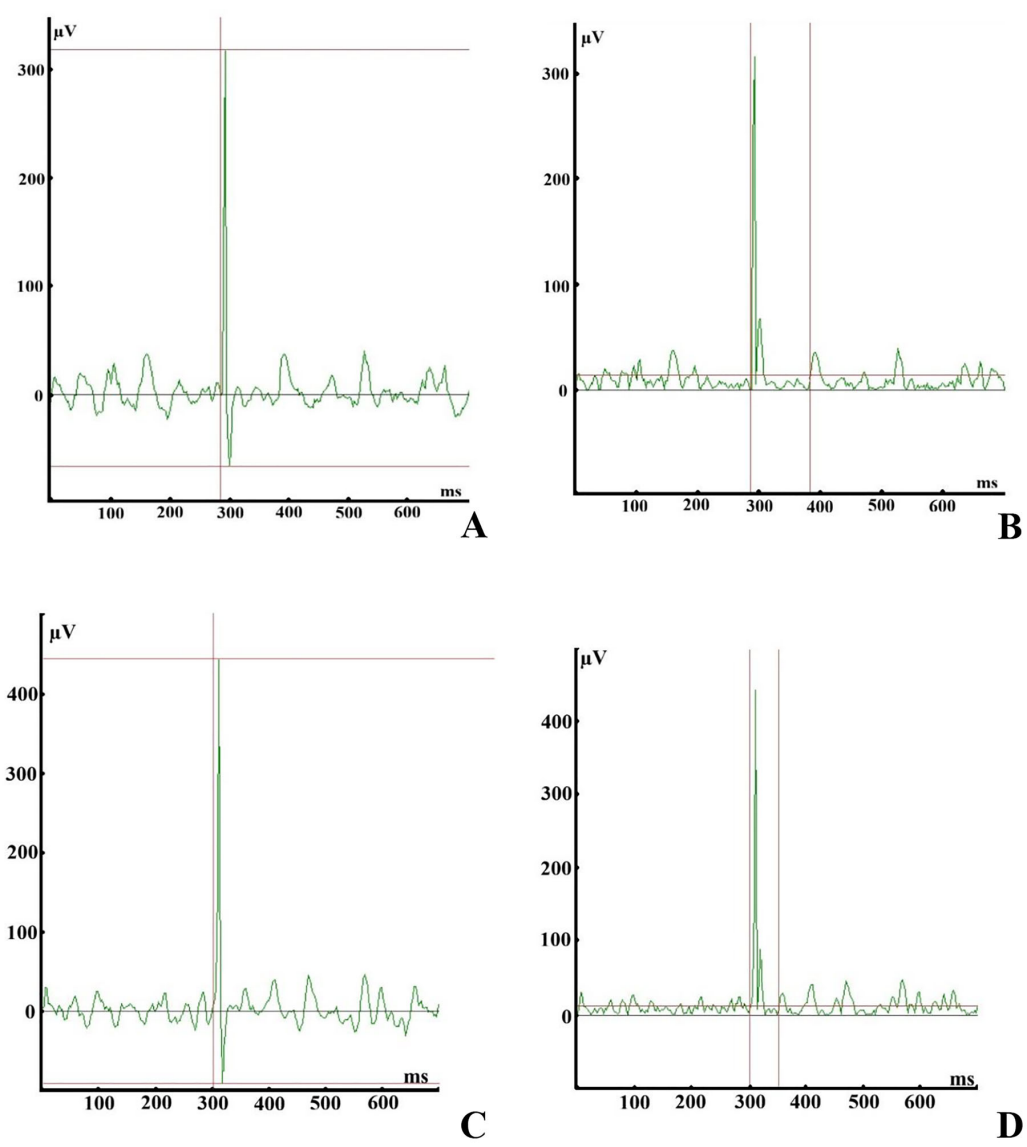
The measurement of (A and C) the motor evoked potential, defined as the amplitude between the horizontal red lines, and (B and D) the cortical silent period, defined as the duration between the vertical red lines, recorded from the peroneus longus muscle at 120% of the active corticomotor threshold across individuals diagnosed with chronic ankle instability, with (C and D) and without (A and B) progressive balance exercises.

In the present study, the electromyographic signal was then rectified and averaged using a root mean square algorithm over a 1‐ms moving window (Futatsubashi and Sekiguchi 2024; Groppa et al. 2012). The CSP was measured from the onset of the MEP to the mean pre‐stimulus background activity plus two standard deviations (Groppa et al. 2012). The moments at which the electromyographic signal exceeded and then returned to the mean pre‐stimulus background electromyographic activity plus two standard deviations, calculated over a 100‐ms period, were identified as the onset and offset of the CSP, respectively (Futatsubashi and Sekiguchi 2024; Groppa et al. 2012; Hupfeld et al. 2020; Škarabot et al. 2019). The mean CSP, measured across 30 trials, is reported in the present study. The methods employed to measure the MEP and the CSP of the peroneus longus muscle at 120% of the ACMT are illustrated in Figure 4.

#### 2.5.2 The Objective Postural Stability Indices

The present study utilized the Biodex balance system (Model 950‐302, Biodex Medical Systems Inc., USA) to assess postural stability control (Figure 2). The platform permits tilting up to 20° in any direction (Jain et al. 2016). In addition, the resistance opposing platform tilt can be adjusted from Level 1, the lowest resistance, to Level 12, the highest resistance (Jain et al. 2016). This system measures the platform's deviation from the reference position along the sagittal axis (*Y*) and the frontal axis (*X*) at a frequency of 20 Hz. The system then computes the anteroposterior stability index, the mediolateral stability index, and the overall stability index using the following equations (Rahnama et al. 2010):

- The anteroposterior stability index = [(∑(0 – *Y*)^2^ / *N*)]^1^/^2^
- The mediolateral stability index = [(∑(0 – *X*)^2^ / *N*)]^1^/^2^
- The overall stability index = [(∑(0 – *Y*)^2^ + ∑(0 – *X*)^2^ / *N*)]^1^/^2^

These stability indices represent the displacement of the center of pressure on the anteroposterior axis, mediolateral axis, and across all axes, respectively (Youssef et al. 2018). The display module provides real‐time feedback on the displacement of the center of pressure for each participant (Youssef et al. 2018).

Participants were instructed to stand barefoot on their dominant foot centered on the platform, lift their opposite foot off the platform with slight hip flexion and 90° knee flexion, flex their dominant knee joint up to 15°, and hold their arms by their sides (Youssef et al. 2018). In addition, participants were required to focus on the display module and attempt to hold the center of pressure at the center of the innermost circle (Abalos and Hung 2022).

To minimize learning effects, the rehearsal trials were limited to one passive trial and one active trial, each lasting 30 s (Rossini and Pauri 2000). In the passive‐rehearsal trial, participants attentively observed the evaluator executing the task. In contrast, in the active‐rehearsal trial, participants autonomously performed the task. Subsequently, three appraisal trials, each lasting 30 s, were executed in random order at resistance Levels 6 and 8, separated by a 30‐s rest interval (Khalili et al. 2022). Any appraisal trial in which lateral trunk flexion or hip abduction exceeded 30°, the opposite limb contacted either the dominant limb or the platform, or a hand grasped the support handle, was repeated in the present study (McKeon et al. 2008; Youssef et al. 2018). The means of the postural stability indices across three trials are reported in the present study.

#### 2.5.3 The Subjective Stability Index

The stability of the ankle can be subjectively assessed in individuals with CAI using the CAIT (Hiller et al. 2006). The validity (the correlation between the Persian version of this tool and the foot and ankle ability measure was greater than 0.41, and the correlation between the Persian version of this tool and the visual analogue scale was greater than 0.64; *p*‐value < 0.05) and reliability (the intraclass correlation coefficient was greater than 0.91; *p*‐value < 0.001) of the Persian version of this tool were established by Hadadi et al. (2017). This tool consists of nine items, each assessing different aspects of ankle stability (Vuurberg et al. 2018). The total score ranges from 0 to 30 (Hiller et al. 2006). Higher scores indicate greater ankle stability (Vuurberg et al. 2018).

### 2.6 Sample Size

In the present study, the CSP of the peroneus longus muscle was considered the primary outcome measure for within‐group and between‐group effects. To estimate the sample size, the present study referenced the study conducted by Chung et al. (2023), who compared the CSP of the soleus muscle at 100% of the ACMT between the intervention group (40.58 ± 20.08 ms) and the control group (78.41 ± 52.68 ms) following one session of balance exercises. Using G*Power software, the present study determined that a minimum of 19 participants per group was required to achieve a 95% confidence level and 80% statistical power.

### 2.7 Statistical Analyses

The present study reported the mean and standard deviation for quantitative variables. In addition, the frequency distributions were presented for qualitative variables, including sex and dominant ankle. The chi‐squared test was used to compare the frequency distribution of sex and dominant ankle between the two groups. The Shapiro–Wilk test was employed to assess the normal distribution of the quantitative variables within each group. According to the results obtained from the Shapiro–Wilk test, either the independent samples *t*‐test or the Mann–Whitney *U* test was used to compare the means of background and dependent variables between the two groups before the intervention. The independent samples *t*‐test was used to compare the means of age, height, weight, time since first and last ankle sprain, and number of giving way episodes in the last 6 months between the two groups. Accordingly, the independent samples *t*‐test was used to compare the means of the RCMT and the CSP between the two groups before progressive balance exercises. However, the Mann–Whitney *U* test was used to compare the medians of the ACMT and the normalized MEP between the two groups before progressive balance exercises. Moreover, the Mann–Whitney *U* test was used to compare the medians of the objective and subjective postural stability indices, including the overall, anteroposterior, and mediolateral stability indices at resistance Levels 6 and 8, and the CAIT score between the two groups before progressive balance exercises. A two‐way analysis of variance was used to evaluate between‐group differences, within‐group changes, and their interaction effects on the corticomotor excitability outcome measures related to the peroneus longus muscle under TMS, including the RCMT, ACMT, normalized MEP, and CSP, as well as on the objective and subjective postural stability indices, including the overall, anteroposterior, and mediolateral stability indices at resistance Levels 6 and 8, and the CAIT score, assessed before and after progressive balance exercises. The present study quantified effect sizes using partial eta‐squared (*η* ^2^ *p*). The intraclass correlation coefficients (ICC_3,4_) with 95% confidence intervals and the standard errors of measurement were calculated to assess both the relative and absolute intra‐rater reliability. The data were analyzed at a significance level of 0.05 using SPSS software.

## 3 Results

There were no significant differences between the two groups in terms of sex (*p*‐value = 0.744), dominant ankle (*p*‐value = 0.631), age (*p*‐value = 0.601), height (*p*‐value = 0.775), weight (*p*‐value = 0.111), and clinical characteristics (*p*‐values > 0.486), as presented in Table 2. Moreover, no significant differences were observed between the two groups in the corticomotor excitability outcome measures under TMS, including the RCMT, ACMT, normalized MEP, and CSP (*p*‐values > 0.222), as well as the objective and subjective postural stability indices, including the overall, anteroposterior, and mediolateral stability indices at resistance Levels 6 and 8, and the CAIT score (*p*‐values > 0.057) before progressive balance exercises.

**TABLE 2:** The frequency distribution for qualitative background variables and the mean and standard deviation for the quantitative background variables and the dependent variables in each group diagnosed with chronic ankle instability.

Variables	Control group (*N* = 19)	Intervention group (*N* = 19)	*p*‐value
Sex (female/male)	11/8	10/9	0.744^a^
	57.9%/42.1%	52.6%/47.4%	
Dominant ankle (left / right)	3/16	2/17	0.631^a^
	15.8%/84.2%	10.5%/89.5%	
Age (year)	37.47 (9.68)	39.21 (10.56)	0.601^b^
Height (cm)	168.74 (7.97)	169.47 (7.82)	0.775^b^
Weight (kg)	75.79 (5.35)	79.53 (8.40)	0.111^b^
Time since first ankle sprain (month)	27.21 (6.05)	28.47 (4.95)	0.486^b^
Time since last ankle sprain (month)	4.92 (1.45)	4.60 (1.47)	0.511^b^
Number of giving way episodes in the last 6 months	5.11 (1.88)	4.74 (1.52)	0.511^b^
Resting corticomotor threshold (% of 2 T)	49.05 (5.96)	51.32 (5.66)	0.238^b^
Active corticomotor threshold (% of 2 T)	44.32 (5.52)	46.53 (5.49)	0.222^c^
Normalized motor evoked potential	2.58 (1.57)	2.78 (1.39)	0.530^c^
Cortical silent period (ms)	78.28 (10.29)	81.41 (7.65)	0.295^b^
Overall Stability Index at resistance Level 8	0.82 (0.24)	0.94 (0.17)	0.065^c^
Anteroposterior Stability Index at resistance Level 8	0.54 (0.17)	0.57 (0.09)	0.178^c^
Mediolateral Stability Index at resistance Level 8	0.48 (0.18)	0.58 (0.13)	0.082^c^
Overall stability index at resistance Level 6	0.84 (0.26)	0.97 (0.20)	0.057^c^
Anteroposterior Stability Index at resistance Level 6	0.62 (0.22)	0.67 (0.14)	0.156^c^
Mediolateral Stability Index at resistance Level 6	0.46 (0.16)	0.50 (0.13)	0.202^c^
Cumberland Ankle Instability Tool score	11.74 (6.39)	10.84 (5.44)	0.815^c^

*Note*: Data are presented as mean (standard deviation).

^a^ The chi‐squared test.

^b^ The independent samples *t*‐test.

^c^ The Mann‐Whitney *U* test were used to statistically compare the two groups at a significance level of ≤ 0.05.

The RCMT and the ACMT corresponding to the peroneus longus muscle under TMS exhibited significant decreases within groups after progressive balance exercises (*p*‐values < 0.001; *η* ^2^ *p* > 0.580), whereas no significant differences were observed between groups (*p*‐values > 0.295; *η* ^2^ *p* < 0.030). However, a significant interaction effect was revealed between the within‐group and the between‐group effects for both the RCMT and the ACMT (*p*‐values < 0.001; *η* ^2^ *p* > 0.623), indicating that these corticomotor thresholds were significantly reduced in the intervention group compared to the control group after progressive balance exercises.

The normalized MEP of the peroneus longus muscle was significantly increased within groups after progressive balance exercises (*p*‐value < 0.001; *η* ^2^ *p* = 0.265), whereas no significant difference was detected between groups (*p*‐value = 0.177; *η* ^2^ *p* = 0.050). Accordingly, the CSP of the peroneus longus muscle showed a significant decrease within groups (*p*‐value < 0.001; *η* ^2^ *p* = 0.749) and a significant difference between groups (*p*‐value < 0.001; *η* ^2^ *p* = 0.307) after progressive balance exercises. Moreover, a significant interaction effect was identified between the within‐group and the between‐group effects for both the normalized MEP and the CSP (*p*‐values < 0.001; *η* ^2^ *p* > 0.311), demonstrating that the intervention group experienced significant changes in the normalized MEP and the CSP compared with the control group after progressive balance exercises.

The objective postural stability indices, including the overall, anteroposterior, and mediolateral stability indices at resistance Levels 6 and 8, were significantly decreased within groups after progressive balance exercises (*p*‐values < 0.001; *η* ^2^ *p* > 0.326), whereas no significant differences were found between groups (*p*‐values > 0.118; *η* ^2^ *p* < 0.066). Accordingly, the subjective postural stability index, assessed by the CAIT score, exhibited a significant increase within groups (*p*‐value < 0.001; *η* ^2^ *p* = 0.420) and a significant difference between groups (*p*‐value = 0.013; *η* ^2^ *p* = 0.158) after progressive balance exercises. Moreover, a significant interaction effect was revealed between the within‐group and the between‐group effects for the objective and subjective postural stability indices (*p*‐values < 0.001; *η* ^2^ *p* > 0.373), indicating that these postural stability indices were significantly improved in the intervention group compared to the control group after progressive balance exercises.

**FIGURE 5:**
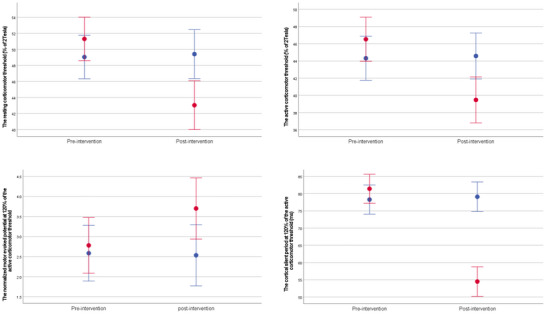
The mean with a 95% confidence interval for the active and resting corticomotor thresholds corresponding to the peroneus longus muscle under transcranial magnetic stimulation, as well as for the normalized motor evoked potential and cortical silent period of the peroneus longus muscle at 120% of the active corticomotor threshold, before and after progressive balance exercises, in the control group (indicated by blue bars) and the intervention group (indicated by red bars).

The results obtained for the corticomotor excitability outcome measures under TMS before and after progressive balance exercises, including the RCMT and the ACMT corresponding to the peroneus longus muscle, as well as the normalized MEP and the CSP of the peroneus longus muscle at 120% of the ACMT, are presented in Table 3 and illustrated in Figure 5. In addition, the findings obtained for the objective and subjective postural stability indices, including the overall, anteroposterior, and mediolateral stability indices at resistance Levels 6 and 8, and the CAIT score, are reported in Table 4.

**TABLE 3:** The means and standard deviations of the corticomotor excitability outcome measures before and after progressive balance exercises in each group diagnosed with chronic ankle instability.

Variables		Pre‐test	Post‐test	*F*	*p*‐value^W^	*η* ^2^ *p*	*F*	*p*‐value^WB^	*η* ^2^ *p*
RCMT (% of 2 T)	CG	49.05 (5.96)	49.42 (6.34)	49.791	**< 0.001**	0.580	59.519	**< 0.001**	0.623
	IG	51.32 (5.66)	43.05 (6.74)						
	F	1.131							
	*p*‐value^B^	0.295							
	*η* ^2^ *p*	0.030							
ACMT (% of 2 T)	CG	44.32 (5.52)	44.58 (5.35)	53.508	**< 0.001**	0.598	62.125	**< 0.001**	0.633
	IG	46.53 (5.49)	39.47 (6.09)						
	*F*	0.672							
	*p*‐value^B^	0.418							
	*η* ^2^ *p*	0.018							
Normalized MEP	CG	2.58 (1.57)	2.53 (1.43)	12.992	**< 0.001**	0.265	16.246	**< 0.001**	0.311
	IG	2.78 (1.39)	3.69 (1.82)						
	*F*	1.896							
	*p*‐value^B^	0.177							
	*η* ^2^ *p*	0.050							
CSP (ms)	CG	78.28 (10.29)	79.09 (8.43)	107.510	**< 0.001**	0.749	121.291	**< 0.001**	0.771
	IG	81.41 (7.65)	54.50 (9.95)						
	*F*	15.928							
	*p*‐value^B^	**< 0.001**							
	*η* ^2^ *p*	0.307							

*Note*: The between‐group effect (denoted by superscript B), the within‐group effect (denoted by superscript W), and the interaction effect between the within‐group and the between‐group effects (denoted by superscript WB) in the analysis of variance (ANOVA) with repeated measures were statistically significant at a *p*‐value ≤ 0.05. Effect sizes are reported using partial eta‐squared (*η* ^2^ *p*). Data are presented as mean (standard deviation). The resting corticomotor threshold (RCMT) and the active corticomotor threshold (ACMT) corresponding to the peroneus longus muscle were assessed under transcranial magnetic stimulation. In addition, the motor evoked potential (MEP) and cortical silent period (CSP) of the peroneus longus muscle were recorded at 120% of the active corticomotor threshold.

The intervention group (IG) practiced progressive balance exercises every other day for 6 weeks, while the control group (CG) continued their daily activities for 6 weeks.

**TABLE 4:** The mean and standard deviation for the objective and subjective stability indices before and after progressive balance exercises in each group diagnosed with chronic ankle instability.

Variables		Pre‐test	Post‐test	*F*	*p*‐value^W^	*η* ^2^ *p*	*F*	*p*‐value^WB^	*η* ^2^ *p*
OSI _at RL 8_	CG	0.82 (0.24)	0.80 (0.22)	67.111	**< 0.001**	0.651	48.573	**< 0.001**	0.574
	IG	0.94 (0.17)	0.62 (0.17)						
	*F*	0.203							
	*p*‐value^B^	0.655							
	*η* ^2^ *p*	0.006							
APSI _at RL 8_	CG	0.54 (0.17)	0.55 (0.15)	51.042	**< 0.001**	0.586	63.375	**< 0.001**	0.638
	IG	0.57 (0.09)	0.37 (0.13)						
	*F*	2.561							
	*p*‐value^B^	0.118							
	*η* ^2^ *p*	0.066							
MLSI _at RL 8_	CG	0.48 (0.18)	0.50 (0.18)	46.006	**< 0.001**	0.561	55.668	**< 0.001**	0.607
	IG	0.58 (0.13)	0.36 (0.14)						
	*F*	0.170							
	*p*‐value^B^	0.683							
	*η* ^2^ *p*	0.005							
OSI _at RL 6_	CG	0.84 (0.26)	0.86 (0.19)	17.444	**< 0.001**	0.326	21.382	**< 0.001**	0.373
	IG	0.97 (0.20)	0.66 (0.24)						
	*F*	0.309							
	*p*‐value^B^	0.582							
	*η* ^2^ *p*	0.009							
APSI _at RL 6_	CG	0.62 (0.22)	0.63 (0.21)	37.091	**< 0.001**	0.507	43.674	**< 0.001**	0.548
	IG	0.67 (0.14)	0.41 (0.19)						
	*F*	1.769							
	*p*‐value^B^	0.192							
	*η* ^2^ *p*	0.047							
MLSI _at RL 6_	CG	0.46 (0.16)	0.47 (0.17)	26.471	**< 0.001**	0.424	38.118	**< 0.001**	0.514
	IG	0.50 (0.13)	0.32 (0.11)						
	*F*	1.537							
	*p*‐value^B^	0.223							
	*η* ^2^ *p*	0.041							
CAIT score	CG	11.74 (6.39)	10.11 (4.09)	26.110	**< 0.001**	0.420	54.120	**< 0.001**	0.601
	IG	10.84 (5.44)	19.89 (6.63)						
	*F*	6.754							
	*p*‐value^B^	**0.013**							
	*η* ^2^ *p*	0.158							

*Note*: The between‐group effect (denoted by superscript B), the within‐group effect (denoted by superscript W), and the interaction effect between the within‐group and the between‐group effects (denoted by superscript WB) in the analysis of variance (ANOVA) with repeated measures were statistically significant at a *p*‐value ≤ 0.05. Effect sizes are reported using partial eta‐squared (*η* ^2^ *p*). Data are presented as mean (standard deviation). The Overall Stability Index (OSI), the Anteroposterior Stability Index (APSI), and the Mediolateral Stability Index (MLSI) were assessed at resistance levels (RLs) 8 and 6. In addition, the subjective stability index was evaluated using the Cumberland Ankle Instability Tool (CAIT). The intervention group (IG) practiced progressive balance exercises every other day for 6 weeks, while the control group (CG) continued their daily activities for 6 weeks.

The present study found excellent intra‐rater reliability for the measurements of corticomotor excitability outcome measures. The intraclass correlation coefficients for these primary outcome measures exceeded 0.800, with the 95% confidence intervals (*p*‐values < 0.001). The standard errors of measurement ranged from 0.287% to 2.870% of 2 T for the RCMT and ACMT, from 0.005 to 0.317 for the normalized MEP, and from 0.132 to 1.306 ms for the CSP.

## 4 Discussion

In the present study, corticomotor excitability corresponding to the peroneus longus muscle were significantly changed in the intervention group compared to the control group following 6 weeks of progressive balance exercises. Moreover, both the objective and subjective stability indices were significantly improved in the intervention group compared to the control group following progressive balance exercises.

Previous studies have reported inconsistent findings regarding corticomotor excitability in individuals with CAI. Studies conducted by Terada et al. (2022), Nanbancha et al. (2019), and Kosik et al. (2017) observed a significant decrease in the MEP of the tibialis anterior and the peroneus longus muscles in individuals with CAI compared to healthy individuals. Accordingly, Pietrosimone and Gribble (2012) reported a significant increase in the RCMT related to the peroneus longus muscle in individuals with CAI compared to healthy individuals. However, Nanbancha et al. (2019) and Terada et al. (2022) found no significant changes in the corticomotor thresholds related to the tibialis anterior and peroneus longus muscles between individuals with and without CAI. The results of the present study cannot be directly compared with those of previous cross‐sectional studies (Kosik et al. 2017; Nanbancha et al. 2019; Pietrosimone and Gribble 2012; Terada et al. 2022). The central nervous system proportionally reweights proprioceptive inputs in sensory cortical areas adjacent to the deprived sensory cortical area following peripheral lesions, which subsequently leads to a reorganization of the corresponding and cooperating areas of the motor cortex (Rossini and Pauri 2000). Accordingly, Kosik et al. (2017) observed a significant decrease in corticomotor area corresponding to the peroneus longus muscle in individuals with CAI. The differences observed in corticomotor excitability across these studies (Nanbancha et al. 2019; Pietrosimone and Gribble 2012; Terada et al. 2022) may be attributed to varying degrees of disruptive neuroplastic changes. Accordingly, Kosik et al. (2017) demonstrated that the corticomotor area representing the peroneus longus muscle may be reduced in proportion to the inversion ankle sprain chronicity. These neuroplastic changes can influence the density of pyramidal neurons and their corresponding excitatory or inhibitory intracortical interneurons in the primary motor area (Nanbancha et al. 2019).

In previous studies, balance control was primarily attributed to cortical level (Chung et al. 2023) or subcortical levels (Beck et al. 2007) following balance exercises, while reflex responses at the spinal level were either attenuated (Chung et al. 2023) or maintained (Beck et al. 2007). The monosynaptic corticospinal pathway, which projects onto α‐motor neurons and Ia afferents, mediates balance control for responses with latencies exceeding 85–100 ms (Taube et al. 2008). This tract facilitates α‐motor neurons and presynaptically inhibits Ia afferents (Taube et al. 2008). Consequently, spinal reflexes, which are inherently destabilizing, can no longer effectively control postural stability (Taube et al. 2008). To enhance ankle stability, the muscles surrounding the ankle joint concurrently activate during a challenging posture (Taube et al. 2008). This synchronized muscle activity provides evidence for the hypothesis that the motor cortex regulates postural stability (Taube et al. 2008).

Previous studies have explored the effects of balance exercises on corticospinal excitability in individuals with (Chung et al. 2023) and without (Bakker et al. 2021; Beck et al. 2007; Hu et al. 2023; Mouthon and Taube 2019) CAI. Bakker et al. (2021) and Hu et al. (2023) found no significant changes in cortical or intracortical activities of the primary motor area following a single session of balance exercises. Neuromuscular adaptation during balance exercises can be facilitated by both repetition (Bakker et al. 2021; Hu et al. 2023; Rossini and Pauri 2000) and external feedback (Hu et al. 2023). These two training principles significantly impact the learning process of balance control. The intervention group in the present study practiced progressive balance exercises every other day for 6 weeks and received real‐time feedback on their center of pressure through a display module. Therefore, the differences observed between the findings of the present study and those reported in previous studies (Bakker et al. 2021; Hu et al. 2023) may be attributed to these two training principles.

Both Beck et al. (2007) and Mouthon and Taube (2019) reported a significant decrease in the MEP of the tibialis anterior muscle following 16 and 6 sessions of balance exercises, respectively. These studies (Beck et al. 2007; Mouthon and Taube 2019) concluded that neural control of postural stability shifts from cortical level to subcortical levels as individuals acquire balance control skills through balance exercises. In contrast, previous studies (Mouthon and Taube 2019; Nandi et al. 2018) have revealed that the demands of controlling complex postures can suppress inhibitory intracortical circuits in the primary motor area, thereby facilitating corticomotor excitability. Therefore, the differences observed in corticomotor excitability between the present study and previous studies (Beck et al. 2007; Mouthon and Taube 2019) may be attributed to varying levels of motor learning.

In contrast to previous studies, Chung et al. (2023) exclusively explored the effects of a single session of balance exercises on corticomotor excitability corresponding to the soleus muscle in individuals with CAI. They found no significant changes in the ACMT and the MEP of the soleus muscle following a single session of balance exercises (Chung et al. 2023). In addition, balance control was not significantly improved following a single session of balance exercises (Chung et al. 2023), while balance control significantly improved in our intervention group following 18 sessions of the progressive balance exercises. The differences in corticomotor excitability between the current study and the study conducted by Chung et al. (2023) may be attributed to differences in the balance exercise protocols and the skill level achieved in balance control in each study.

The CSP reflects a temporarily interrupted electromyographic activity of the target muscle, which occurs subsequent to a MEP elicited by TMS targeting the primary motor cortex (Škarabot et al. 2019; Zeugin and Ionta 2021). The corticospinal and corticosubcortical pathways may concurrently contribute to the CSP (Hupfeld et al. 2020; Škarabot et al. 2019; Zeugin and Ionta 2021). The central nervous system selectively suppresses excessive excitatory inputs during this period to ensure precise motor control and to reduce conflicting motor outputs (Hupfeld et al. 2020; Škarabot et al. 2019; Zeugin and Ionta 2021). The corticospinal pathway predominantly modulates the first component of the CSP, lasting approximately 50 to 80 ms, which is mediated through recurrent inhibition of motor neurons by Renshaw cells, monosynaptic disfacilitation of motoneurons due to reduced Ia drive, disynaptic inhibition of motoneurons by inhibitory interneurons due to enhanced Ib drive, and afterhyperpolarization of motor neurons (Hupfeld et al. 2020; Škarabot et al. 2019; Zeugin and Ionta 2021). Accordingly, the hyperdirect and indirect pathways, mediated by the gamma‐aminobutyric acid neuromodulator within the cortico‐basal ganglia‐thalamo‐cortical loop, may interactively regulate the last component of the CSP, extending from approximately 50–80 ms up to 300 ms (Hupfeld et al. 2020; Škarabot et al. 2019; Zeugin and Ionta 2021). Based on the MEP duration of 29.4 ms reported in healthy participants by Brum et al. (2016), our CSP findings following progressive balance exercises were effectively confined to the first component of the CSP and were predominantly mediated by corticospinal pathways, likely reflecting the mechanisms outlined below. The figure‐of‐eight coil focally stimulates the primary motor cortex, which may suppress inhibitory pathways from the supplementary or premotor cortices (Hupfeld et al. 2020). In addition, learning balance control under cognitively challenging conditions enhances frontal cortex excitability, which may subsequently attenuate inhibitory pathways across the corticosubcortical pathway (Zeugin and Ionta 2021).

Previous studies have demonstrated that cortical structures predominantly regulate postural stability during the early stages of learning (Beck et al. 2007). To automate balance control, subcortical structures gradually assume a more prominent role in regulating postural stability, leading to a decrease in corticomotor excitability (Beck et al. 2007; Hu et al. 2023; Mouthon and Taube 2019). The present study implemented a protocol of progressive balance exercises with concurrent real‐time visual feedback. In contrast to the present study, previous studies have primarily relied on non‐progressive balance exercises (Bakker et al. 2021; Chung et al. 2023; Hu et al. 2023; Mouthon and Taube 2019) without providing any feedback during training (Beck et al. 2007; Chung et al. 2023; Hu et al. 2023; Mouthon and Taube 2019). Consequently, a sustained increase in corticomotor excitability observed following progressive balance exercises in the present study may be attributed to the continued experience of motor learning challenges throughout the 6‐week intervention (Hupfeld et al. 2020; Rossini and Pauri 2000).

Previous studies that explored the impact of balance exercises on corticomotor excitability have either neglected to assess balance control (Beck et al. 2007) or employed rudimentary and perfunctory assessment methods (Bakker et al. 2021; Mouthon and Taube 2019). Studies that precisely measured balance indices found no significant changes following balance exercises (Bakker et al. 2021; Chung et al. 2023). Their results are not unexpected, as participants received only a single session of balance exercises (Bakker et al. 2021; Chung et al. 2023). In the present study, both the objective and subjective stability indices significantly improved after 6 weeks of balance exercises. In contrast to previous studies, the present study identified a significant link between corticomotor excitability and postural stability control following 6 weeks of balance exercises (Bakker et al. 2021; Chung et al. 2023).

### 4.1 Methodological Considerations

Previous studies have primarily explored corticomotor excitability related to the muscles controlling movements in the sagittal plane following balance exercises (Bakker et al. 2021; Beck et al. 2007; Chung et al. 2023; Hu et al. 2023; Mouthon and Taube 2019). However, individuals experiencing recurrent inversion ankle sprains commonly exhibit proprioceptive deficits (Ha et al. 2018) and muscular dysfunction (Plangtaisong et al. 2021) in the frontal plane. Therefore, the present study specifically explored corticomotor excitability related to the peroneus longus muscle, a primary contributor to ankle joint stability in the frontal plane (Nandi et al. 2018; Needle et al. 2013), to comprehensively evaluate neuromuscular adaptations following progressive balance exercises in individuals with CAI. Therefore, methodological differences may account for the discrepancies observed between the findings of the present study and those reported in previous study (Chung et al. 2023).

Corticomotor neuron pools demonstrate considerable output variability across different intensities of supra‐threshold stimulation and under various physiological conditions (Bachasson et al. 2016; Krile et al. 2023). Most previous studies have employed a range of stimulation intensities (Beck et al. 2007; Chung et al. 2023; McLeod et al. 2015; Terada et al. 2022) to comprehensively assess corticomotor excitability while avoiding saturation effects (Beck et al. 2007). The inconsistent findings observed among previous studies on corticomotor excitability may be attributed to the varying ranges of stimulus intensity employed (Krile et al. 2023). Collectively, there is no established consensus on the ideal range of stimulus intensities for assessing corticomotor excitability (Bachasson et al. 2016; Sivaramakrishnan and Madhavan 2020).

Various methods have been previously employed to normalize the MEPs, including normalization to the electromyographic amplitude of background voluntary muscle contraction (Beck et al. 2007; Nandi et al. 2018; Terada et al. 2022) or maximal voluntary muscle contraction (Dharia et al. 2021), maximal motor amplitude evoked by corticomotor stimulation (Needle et al. 2013), and maximal muscle amplitude elicited by peripheral nerve stimulation (Beck et al. 2007; Chung et al. 2023; Hu et al. 2023; Kosik et al. 2017; McLeod et al. 2015; Terada et al. 2016). Currently, there is no consensus regarding the most reliable normalization method (Dharia et al. 2021). However, Dharia et al. (2021) reported that normalizing the motor evoked amplitude to the electromyographic amplitude of maximal voluntary muscle contraction provides more reliable results compared to normalizing to the maximal motor amplitude evoked by corticomotor stimulation.

The double‐cone coil generates a stronger magnetic field compared to the figure‐of‐eight coil, resulting in more intense and less focused stimulation of deeper areas of the brain (Hupfeld et al. 2020; Schecklmann et al. 2020). Therefore, the figure‐of‐eight coil may be used to precisely stimulate the primary motor cortex areas corresponding to both the lower limb muscles, located superficially and medially on the precentral gyrus, and the upper limb muscles, located superficially and laterally on the precentral gyrus (Hupfeld et al. 2020; Kandel et al. 2021). Based on the findings of Schecklmann et al. (2020), the results observed for the corticomotor threshold in the present study correspond to those reported by Needle et al. (2013) and Terada et al. (2016, 2022), whereas they conflict with those reported by McLeod et al. (2015), Nanbancha et al. 2019, Pietrosimone and Gribble (2012). These differences may be attributed to methodological differences between the present study and previous studies. The corticomotor threshold depends on various factors, including the maximum output of the magnetic stimulator, the task, the target muscle, the background activity level of the muscle, the method employed to define the corticomotor threshold, and the position, orientation, and structural design characteristics of the coil, such as the angle, diameter, and density of the windings (Hupfeld et al. 2020; Schecklmann et al. 2020). These factors have been either reported incompletely (Chung et al. 2023; Kosik et al. 2017; Nanbancha et al. 2019; Needle et al. 2013; Terada et al. 2022) or addressed inconsistently (Chung et al. 2023; McLeod et al. 2015; Needle et al. 2013; Pietrosimone and Gribble 2012; Terada et al. 2016, 2022) in previous research, which hinders the direct comparison of corticomotor threshold values across studies.

### 4.2 Limitations and Suggestions

Ideally, corticomotor excitability should be evaluated during single‐leg stance following progressive balance exercises (Taube et al. 2008). However, the present study, consistent with previous studies (Kosik et al. 2017; McLeod et al. 2015; Nanbancha et al. 2019), assessed corticomotor excitability in a seated position to avoid the influence of confounding variables associated with single‐leg stance, including background muscle activity (Chung et al. 2023; Nandi et al. 2018), muscle excursion (Dick et al. 2024; Terada et al. 2022), and muscle fatigue (Dick et al. 2024).

A passive control group was incorporated into the present study, consistent with a closely related previous study (Chung et al. 2023), to provide a comparative baseline for evaluating the impact of progressive balance exercises on corticomotor excitability. Accordingly, the risk of bias influencing subjective outcome measure cannot be disregarded in the present study. Therefore, future studies should incorporate an active control group to investigate the effects of progressive balance exercises on corticomotor excitability.

A follow‐up assessment was not incorporated in the present study. Accordingly, this study cannot definitively establish the long‐term impact of progressive balance exercises on corticomotor excitability. Therefore, future studies should replicate the present study with a follow‐up assessment to investigate these long‐term effects.

Moreover, future studies should concurrently explore the effects of progressive balance exercises on the excitability of the primary motor cortex and spinal cord corresponding to the peroneus longus muscle to identify the extent to which the corticospinal and corticosubcortical pathways contribute to the CSP.

Adverse structural and functional changes in the peripheral and central nervous systems, as well as in the connective tissues, impair balance control in older adults (Henry and Baudry 2019). These changes may develop insidiously during middle age with no apparent functional or clinical evidence. Therefore, the results obtained in the present study, which included young and middle‐aged adults, should be interpreted cautiously.

The results obtained in the present study may be generalized to individuals with CAI who achieve postural stability control following 6 weeks of the progressive balance exercises.

## 5 Conclusions

The present study revealed, for the first time, that 6 weeks of progressive balance exercises significantly increased corticomotor excitability corresponding to the peroneus longus muscle and improved balance control in individuals suffering from CAI.

## Author Contributions

All authors contributed to this study, and approved the final version of the manuscript.

## Funding

This study was supported by the Semnan University of Medical Sciences under Grant Number: 2093.

## Conflicts of Interest

The authors declare no conflicts of interest.

## Data Availability

Data are available on request from the authors.
